# A Dataset of Amphibian Species in U.S. National Parks

**DOI:** 10.1038/s41597-023-02836-2

**Published:** 2024-01-04

**Authors:** Benjamin J. LaFrance, Andrew M. Ray, Robert N. Fisher, Evan H. Campbell Grant, Charles Shafer, David A. Beamer, Stephen F. Spear, Todd W. Pierson, Jon M. Davenport, Matthew L. Niemiller, R. Alexander Pyron, Brad M. Glorioso, William J. Barichivich, Brian J. Halstead, Kory G. Roberts, Blake R. Hossack

**Affiliations:** 1 Northern Rockies Conservation Cooperative, Jackson, WY 83001 USA; 2 National Park Service—Greater Yellowstone Network, Bozeman, MT 59715 USA; 3 National Park Service—Southern Plains Network, Pecos, NM 87552 USA; 4 U.S. Geological Survey—Western Ecological Research Center, San Diego, CA 92101 USA; 5 U.S. Geological Survey—Eastern Ecological Research Center (Patuxent Wildlife Research Center), Turners Falls, MA 01376 USA; 6 Office of Research, Economic Development and Engagement, East Carolina University, Greenville, NC 27858 USA; 7 U.S. Geological Survey—Upper Midwest Environmental Sciences Center, La Crosse, WI 54603 USA; 8 Department of Ecology, Evolution, and Organismal Biology, Kennesaw State University, Kennesaw, GA 30144 USA; 9 Department of Biology, Appalachian State University, Boone, NC 28608 USA; 10 Department of Biological Sciences, The University of Alabama in Huntsville, Huntsville, AL 35899 USA; 11 Department of Biological Sciences, The George Washington University, Washington, DC 20052 USA; 12 Department of Vertebrate Zoology, National Museum of Natural History Smithsonian Institution, Washington, DC 20560 USA; 13 U.S. Geological Survey—Wetland and Aquatic Research Center, Lafayette, LA 70506 USA; 14 U.S. Geological Survey—Wetland and Aquatic Research Center, Gainesville, FL 32653 USA; 15 U.S. Geological Survey—Western Ecological Research Center, Dixon, CA 95620 USA; 16 Arkansas Herpetological Atlas, Bella Vista, AR 72715 USA; 17 U.S. Geological Survey—Northern Rocky Mountain Science Center; Wildlife Biology Program, University of Montana, Missoula, MT 59812 USA

**Keywords:** Herpetology, Biodiversity

## Abstract

National parks and other protected areas are important for preserving landscapes and biodiversity worldwide. An essential component of the mission of the United States (U.S.) National Park Service (NPS) requires understanding and maintaining accurate inventories of species on protected lands. We describe a new, national-scale synthesis of amphibian species occurrence in the NPS system. Many park units have a list of amphibian species observed within their borders compiled from various sources and available publicly through the NPSpecies platform. However, many of the observations in NPSpecies remain unverified and the lists are often outdated. We updated the amphibian dataset for each park unit by collating old and new park-level records and had them verified by regional experts. The new dataset contains occurrence records for 292 of the 424 NPS units and includes updated taxonomy, international and state conservation rankings, hyperlinks to a supporting reference for each record, specific notes, and related fields which can be used to better understand and manage amphibian biodiversity within a single park or group of parks.

## Background & Summary

With habitat loss as a major driver decreasing biodiversity, protected areas are increasingly essential to conservation^1–4^. The U.S. National Park Service (NPS) manages a wide variety of lands protected from development, overuse, overharvesting, and other potentially impactful activities. Although most NPS units were established to protect historical, cultural, or geologically unique features, these protected park units can also be important for conservation of species such as amphibians^5,6^. The unusual geologic and natural features that characterize some national parks and protected areas likely contribute to the presence of endemic species or distinct populations^7,8^. To better understand how NPS lands contribute to amphibian diversity in the U.S. (Table 1), we updated a dataset of amphibian species occurrence in each park unit that had records in NPSpecies.

**Table 1:** Number of amphibian species documented in the U.S.A. and on National Park Service (NPS) lands, categorized by International Union for Conservation of Nature (IUCN) Red List Index^11^.

IUCN status	No. of species in USA	No. of species on NPS land	Percent of species documented on NPS land
Least Concern	186	158	85.0
Near Threatened	36	17	47.2
Vulnerable	33	10	30.3
Endangered	20	6	30.0
Critically Endangered	7	0	0.0
Extinct in the Wild	1	0	0.0
Extinct	2	0	0.0
Data Deficient	69	39	56.6
Total	354	230	65.0

As a starting point for our updated dataset, we began with the amphibian data available from the NPSpecies platform, an NPS multi-taxa database of species observations in national park units^9^. The associated metadata in the original NPSpecies database included a 4-letter park code to denote where the species observation occurred, the species taxonomy (filled in at each park’s discretion without following uniform taxonomy), “GRank” and “SRank” based on NatureServe status (over a third of the records had no data in this column), a nativeness column, as well as other fields such as “ozone” which is important for other species monitored by the NPS (such as ozone sensitive plants) but is extraneous for this dataset. Information about the specific date of observations in NPSpecies is limited.

**Fig. 1:**
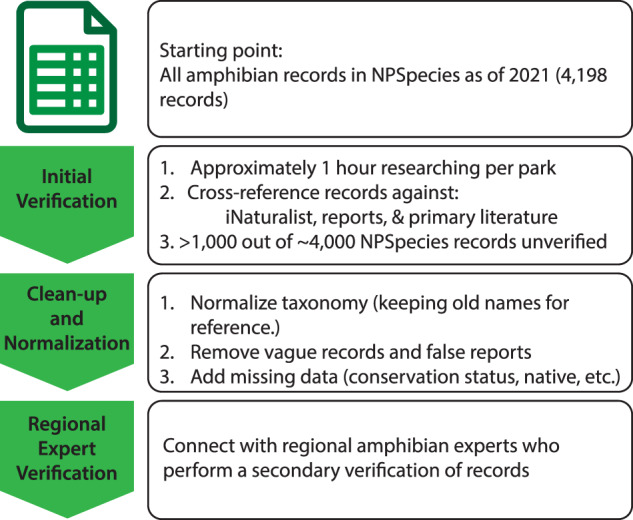
Workflow used to generate an updated dataset of amphibian occurrence for park units within the U.S. National Park Service.

As of 01 March 2021, NPSpecies had 4,198 records of amphibian species across all park units. Although NPSpecies is internally validated, over 1,000 of the records were still listed as unconfirmed or unverified. We used available nomenclature in the Integrated Taxonomic Information System (ITIS) to provide a common taxonomy for consistency and comparability^10,11^. We also cleaned the NPSpecies list by removing 836 unverifiable park-level species occurrences and adding 115 new occurrences, changing occurrence status or taxonomy on over 1,000 records, and had regional subject matter experts verify the updated records (Fig. 1). As an example of changing occurrence status and cleaning the data, Death Valley National Park had 71 amphibian species listed in NPSpecies, but only 10 species were verified as Present, Adjacent, or even Possibly Present. A list of all associated data and definitions (such as what Present, Adjacent or Possible mean) for each record are in Table 2^12^. The 115 new records were added opportunistically when references or regional subject matter experts that verified an original record (see below) had additional information about species or park records not yet documented in parks^13^. No additional data sources (e.g., HerpMapper or iNaturalist) were used for adding new species during this initial dataset revision.

**Table 2:** Column headers and additional information to interpret their significance in the dataset of amphibian occurrence.

Column Heading	Description
Park_Code	The 4-letter acronym to identify the specific park unit
Park_Name	Full name of the national park unit
IM_Network	The 4-letter acronym for the associated NPS Inventory and Monitoring Network
CASC_Region	The 2-letter acronym for the associated Climate Adaptation Science Center region
State	The US state that the specific park lies within (for the centroid of the park)
TSN	Taxonomic Serial Number, unique identifier for each amphibian species in accordance with ITIS
Order	Taxonomic order of amphibians for the species (Anura or Caudata)
Family	Taxonomic family of the species
Scientific_Name	Scientific name for the amphibian (*Genus* + *specific epithet*)
Common_Names	Common names used for the species
Verified_Date	The date when the observation was verified (ISO standard format of YYYY-MM-DD)
Park_OccurrenceStatus	Verified status in park—Present, Possible, Adjacent, Historic, No; updated from NPSpecies “Occurrence” field also included
	Present = the species is present within this park unit
	Possible = the species is possibly found on this park unit but has not been confirmed
	Adjacent = the species has been found on land near but not within this park unit (often within ~50 km)
	Historic = this species has been observed in this park in the past (before the year 2000), but is unlikely to be found now
	No = this species has not been observed in this park based on our research
Notes	Notes included during the verification process (includes both primary and secondary verification)
Verified_Source	Link or note regarding reference material (report, primary literature, etc.)
Park_Synonyms	Alternative scientific name (outdated, ITIS invalid, subspecies, or park preferred name)
NPSpecies_Occurrence	Present, Probably Present, Unconfirmed, Not in Park (designation in 2021 version of NPSpecies)
NPSpecies_OccTag	More information regarding occurrence (Historical; False Report) from the 2021 version of NPSpecies
Abundance	NPS ranking akin to IUCN Status—Abundant, Common, Occasional, Uncommon, Rare, Unknown
Nativeness	Whether species is native, non-native, or unknown
GRank	Global Conservation Status Rank (data from www.natureserve.org)
SRank	Sub-national rank for the species (field indicates the state by two-letter code, followed by the status)
IUCNRank	The conservation status of the species as defined on the International Union for Conservation of Nature (IUCN) Red List

Many of these columns can also be found in the NPSpecies User Guide^12^.

**Fig. 2:**
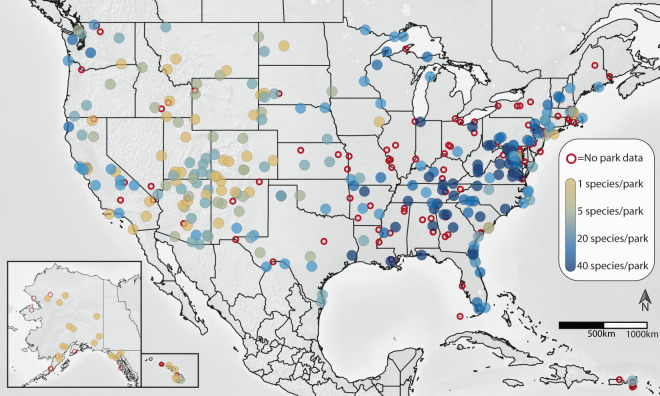
Amphibian species richness (tan to blue gradient) for U.S. National Park Service units (centroids), based on records in the new dataset. Empty red circles denote a park unit lacking any amphibian records in the updated dataset. Alaska and Hawaii are not drawn to scale.

Overall, the updated dataset accounts for approximately 70% of the units managed by the NPS (Fig. 2), and only includes those parks originally present within the 2021 version of NPSpecies dataset. Based on species lists from AmphibiaWeb, International Union for Conservation of Nature (IUCN), the USGS National Amphibian Atlas (as of 08 May 2023)^14,15^, approximately 65% of the amphibian species documented in the U.S. were found in NPS managed areas (230 of 354; Table 1). A few species (mostly *Eleutherodactylus* and *Desmognathus*) not listed in any of the above sources, but which have verified occurrences from published sources were included in the dataset^13^. As with any national-scale project with ongoing efforts, this list is not exhaustive and some species that might actually or possibly exist on or near NPS lands may not be included. Similarly, the dynamic status and uncertainty around taxonomic classification for some species, such as many frogs in the family Hylidae and salamanders in the *Desmognathus* and *Plethodon* genera, likely contributes a small amount of error or ephemerality to the new dataset. Also, there are additional resources for amphibian data in the U.S. (e.g., iNaturalist, GBIF, HerpMapper) that may provide updates to the NPSpecies data archive^16,17^. Future efforts may focus on the integration of more complete occurrence records from all NPS units with managed lands and other sources.

Based on the IUCN Red List status (http://www.iucnredlist.org), the updated dataset indicates that the U.S. National Park System under-represents rare or imperiled species. As an example, 85% of the amphibian species of Least Concern are represented in the dataset. In contrast, only 47% of near-threatened and 30% of endangered amphibians in the U.S. are in the current dataset (Table 1).

The final verified dataset has been deposited as a publicly available NPS DataStore Project under the Integrated Resource Management Applications Portal (10.57830/2301647)^18^.

## Methods

### Data collection

The final dataset was built from initial data downloaded from NPSpecies^9^, which consisted of 4,198 amphibian records as of 01 March 2021. We performed an initial validation which consisted of spending approximately 1 hour per park unit cross-checking the NPSpecies data against primary literature, reports, theses, range maps^19^, and verified iNaturalist observations. Each record was given a hyperlink to a reference as well as any relevant notes about the record or citation. After initial verification, the dataset was taxonomically normalized in accordance with ITIS^11^. All subspecies designations and non-standard nomenclature was retained in the “ParkSynonyms” field.

Records which lacked specificity (only family- or genus-level information provided) were deleted, as well as any obviously false or unverifiable observations (e.g., where a species was recorded well outside of its published range). Upon taxonomic normalization and initial dataset cleaning, we contacted regional subject matter experts to perform a final verification and comment on observations specific to their region. Finally, each record was assigned a conservation status. The global rank (GRank) and state rank (SRank) were based on NatureServe data^20^, as well as a the previously mentioned status based on the IUCN Red List^15^. To aid in management, each dataset entry was also given a field to denote its assignment to one of the 32 NPS Inventory and Monitoring Networks^21^.

## Data Records

The verified dataset maintains a similar format to that represented in NPSpecies, comprised of a single CSV file. Each row of the spreadsheet indicates a unique park-level species occurrence record, while each column heading provides information about that record. Information about each column is given in Table 2. The dataset is available at NPS DataStore (10.57830/2301647)^18^.

## Technical Validation

The verified dataset underwent considerable technical validation. Initial verification was performed by the first two authors of this manuscript, spending approximately 1 hour per park obtaining references for each record within the dataset. Next, the following steps were used to improve dataset quality and comparability: (1) records with “absent” occurrence data and no verifiable references to the contrary were removed, as absent data can be misleading and are rarely reported in occurrence datasets; (2) records with no species-level information were removed; (3) record taxonomy was normalized to ITIS valid species names as of 2022; (4) any missing information for each original record was added for completeness (i.e., some original records were missing values in fields such as nativeness, GRank, SRank, and common names); and (5) species occurrence records were cross-checked with published range maps^19^. Range maps often overestimate species distributions. Any park with species records outside the known range map was scrutinized to either reclassify the species to accurately reflect the range or remove the record entirely. For example, most NPS park units in the western United States still list *Ambystoma tigrinum* as the tiger salamander species present even though the western tiger salamander (*A. mavortium*) was described as a distinct species in 1996^22^.

**Fig. 3:**
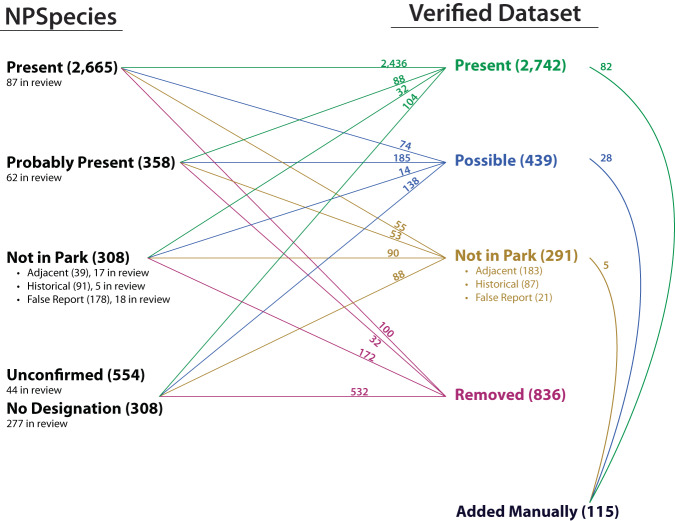
A web diagram comparing the classification status of amphibian occurrence records from the 2021 version of NPSpecies (left; https://irma.nps.gov/npspecies/) to the final, verified, and updated amphibian dataset (right; 10.57830/2301647).

As a final technical verification, regional subject matter experts were asked to provide comments and verify each record relevant to their geography. As a final check, the verified dataset was compared back to the original NPSpecies records, noting all discrepancies and changes (Fig. 3). For example, for the 2,665 species occurrence records in NPSpecies “Present” category, 2,436 were also classified as *Present* within the verified, updated dataset. However, for the remaining *Present* records in the updated dataset, 88 records were originally classified as *Possibly Present*, 32 records were originally classified as *Not in the Park*, 104 records were originally unclassified (either unconfirmed or not given a designation), and 82 new records were added. Also, instead of the original intermediate classification of *Probably Present*, which indicates some significant likelihood that is difficult to represent by occurrence data, we use the more neutral term *Possible*. All these updates and technical validations align with best practices employed in other large occurrence datasets^23–25^.

## Data Availability

No custom code was used to generate or process the data described in this manuscript.
