# Publisher Correction: A Dataset of Amphibian Species in U.S. National Parks

**DOI:** 10.1038/s41597-024-03257-5

**Published:** 2024-04-22

**Authors:** Benjamin J. LaFrance, Andrew M. Ray, Robert N. Fisher, Evan H. Campbell Grant, Charles Shafer, David A. Beamer, Stephen F. Spear, Todd W. Pierson, Jon M. Davenport, Matthew L. Niemiller, R. Alexander Pyron, Brad M. Glorioso, William J. Barichivich, Brian J. Halstead, Kory G. Roberts, Blake R. Hossack

**Affiliations:** 1 Northern Rockies Conservation Cooperative, Jackson, WY 83001 USA; 2 National Park Service—Greater Yellowstone Network, Bozeman, MT 59715 USA; 3 National Park Service—Southern Plains Network, Pecos, NM 87552 USA; 4 U.S. Geological Survey—Western Ecological Research Center, San Diego, CA 92101 USA; 5 U.S. Geological Survey—Eastern Ecological Research Center (Patuxent Wildlife Research Center), Turners Falls, MA 01376 USA; 6 Office of Research, Economic Development and Engagement, East Carolina University, Greenville, NC 27858 USA; 7 U.S. Geological Survey—Upper Midwest Environmental Sciences Center, La Crosse, WI 54603 USA; 8 Department of Ecology, Evolution, and Organismal Biology, Kennesaw State University, Kennesaw, GA 30144 USA; 9 Department of Biology, Appalachian State University, Boone, NC 28608 USA; 10 Department of Biological Sciences, The University of Alabama in Huntsville, Huntsville, AL 35899 USA; 11 Department of Biological Sciences, The George Washington University, Washington, DC 20052 USA; 12 Department of Vertebrate Zoology, National Museum of Natural History Smithsonian Institution, Washington, DC 20560 USA; 13 U.S. Geological Survey—Wetland and Aquatic Research Center, Lafayette, LA 70506 USA; 14 U.S. Geological Survey—Wetland and Aquatic Research Center, Gainesville, FL 32653 USA; 15 U.S. Geological Survey—Western Ecological Research Center, Dixon, CA 95620 USA; 16 Arkansas Herpetological Atlas, Bella Vista, AR 72715 USA; 17 U.S. Geological Survey—Northern Rocky Mountain Science Center; Wildlife Biology Program, University of Montana, Missoula, MT 59812 USA

**Keywords:** Herpetology, Biodiversity

Correction to: *Scientific Data* 10.1038/s41597-023-02836-2, published online 04 January 2024

The copyright holder for this article was incorrectly given as ‘This is a U.S. Government work and not under copyright protection in the US; foreign copyright protection may apply’ but should have been ‘The Author(s)’. The original article has been corrected.

